# Transactions of the Minnesota State Homœpathic Institute

**Published:** 1882-12

**Authors:** 


### BOOK NOTICES, REVIEWS, ETC.

Transactions of the Minnesota State Homœopathic Institute. Years, 1867–1882. Drs. A. A. Camp, Wm. E. Leonard, and C. W. Crary, Publishing Committee. Minneapolis, 1882.

Through the courtesy of Dr. Camp we have a copy of the Transactions of this Institute. This is the first endeavor of a young society to collect its papers in book-form, and as such is very creditable.

Some of the papers are very interesting and instructive. We wish the institute a successful future.

